# Serosurvey for SARS-CoV-2 among blood donors in Wuhan, China from September to December 2019

**DOI:** 10.1093/procel/pwac013

**Published:** 2022-05-24

**Authors:** Le Chang, Lei Zhao, Yan Xiao, Tingting Xu, Lan Chen, Yan Cai, Xiaojing Dong, Conghui Wang, Xia Xiao, Lili Ren, Lunan Wang

**Affiliations:** National Center for Clinical Laboratories, Beijing Hospital, National Center of Gerontology, Institute of Geriatric Medicine, Chinese Academy of Medical Sciences, Beijing 100730, China; Beijing Engineering Research Center of Laboratory Medicine, Beijing Hospital, Beijing 100730, China; Department of Laboratory, Wuhan Blood Center, Wuhan 430030, China; Confirmation Laboratory for Transfusion Transmitted Disease, Institute of Blood Transfusion of Hubei Province, Wuhan 430030, China; National Health Commission of the People’s Republic of China Key Laboratory of Systems Biology of Pathogens, Institute of Pathogen Biology, Chinese Academy of Medical Sciences and Peking Union Medical College, Beijing 100730, China; Key Laboratory of Respiratory Disease Pathogenomics, Chinese Academy of Medical Sciences and Peking Union Medical College, Beijing 100730, China; Department of Laboratory, Wuhan Blood Center, Wuhan 430030, China; Confirmation Laboratory for Transfusion Transmitted Disease, Institute of Blood Transfusion of Hubei Province, Wuhan 430030, China; National Health Commission of the People’s Republic of China Key Laboratory of Systems Biology of Pathogens, Institute of Pathogen Biology, Chinese Academy of Medical Sciences and Peking Union Medical College, Beijing 100730, China; Key Laboratory of Respiratory Disease Pathogenomics, Chinese Academy of Medical Sciences and Peking Union Medical College, Beijing 100730, China; Department of Quality Control, Wuhan Blood Center, Wuhan 430030, China; National Health Commission of the People’s Republic of China Key Laboratory of Systems Biology of Pathogens, Institute of Pathogen Biology, Chinese Academy of Medical Sciences and Peking Union Medical College, Beijing 100730, China; Key Laboratory of Respiratory Disease Pathogenomics, Chinese Academy of Medical Sciences and Peking Union Medical College, Beijing 100730, China; National Health Commission of the People’s Republic of China Key Laboratory of Systems Biology of Pathogens, Institute of Pathogen Biology, Chinese Academy of Medical Sciences and Peking Union Medical College, Beijing 100730, China; Key Laboratory of Respiratory Disease Pathogenomics, Chinese Academy of Medical Sciences and Peking Union Medical College, Beijing 100730, China; National Health Commission of the People’s Republic of China Key Laboratory of Systems Biology of Pathogens, Institute of Pathogen Biology, Chinese Academy of Medical Sciences and Peking Union Medical College, Beijing 100730, China; Key Laboratory of Respiratory Disease Pathogenomics, Chinese Academy of Medical Sciences and Peking Union Medical College, Beijing 100730, China; National Health Commission of the People’s Republic of China Key Laboratory of Systems Biology of Pathogens, Institute of Pathogen Biology, Chinese Academy of Medical Sciences and Peking Union Medical College, Beijing 100730, China; Key Laboratory of Respiratory Disease Pathogenomics, Chinese Academy of Medical Sciences and Peking Union Medical College, Beijing 100730, China; National Center for Clinical Laboratories, Beijing Hospital, National Center of Gerontology, Institute of Geriatric Medicine, Chinese Academy of Medical Sciences, Beijing 100730, China; Beijing Engineering Research Center of Laboratory Medicine, Beijing Hospital, Beijing 100730, China; Graduate School, Peking Union Medical College, Chinese Academy of Medical Sciences, Beijing 100730, China

**Keywords:** severe acute respiratory syndrome coronavirus 2, coronavirus disease 2019, seroprevalence, neutralizing antibodies, blood donors

## Abstract

The emerging of severe acute respiratory syndrome coronavirus 2 (SARS-CoV-2) caused COVID-19 pandemic. The first case of COVID-19 was reported at early December in 2019 in Wuhan City, China. To examine specific antibodies against SARS-CoV-2 in biological samples before December 2019 would give clues when the epidemic of SARS-CoV-2 might start to circulate in populations. We obtained all 88,517 plasmas from 76,844 blood donors in Wuhan between 1 September and 31 December 2019. We first evaluated the pan-immunoglobin (pan-Ig) against SARS-CoV-2 in 43,850 samples from 32,484 blood donors with suitable sample quality and enough volume. Two hundred and sixty-four samples from 213 donors were pan-Ig reactive, then further tested IgG and IgM, and validated by neutralizing antibodies against SARS-CoV-2. Two hundred and thirteen samples (from 175 donors) were only pan-Ig reactive, 8 (from 4 donors) were pan-Ig and IgG reactive, and 43 (from 34 donors) were pan-Ig and IgM reactive. Microneutralization assay showed all negative results. In addition, 213 screened reactive donors were analyzed and did not show obviously temporal or regional tendency, but the distribution of age showed a difference compared with all tested donors. Then we reviewed SARS-CoV-2 antibody results from these donors who donated several times from September 2019 to June 2020, partly tested in a previous published study, no one was found a significant increase in S/CO of antibodies against SARS-CoV-2. Our findings showed no SARS-CoV-2-specific antibodies existing among blood donors in Wuhan, China before 2020, indicating no evidence of transmission of COVID-19 before December 2019 in Wuhan, China.

## Introduction

Severe acute respiratory syndrome coronavirus 2 (SARS-CoV-2) was first identified in December 2019 (Ren et al., 2020; Zhu et al., 2020), then tested and reported positive cases in many parts of the world. The novel beta-coronavirus could transmit via respiratory droplets and close contact and mainly infect bronchial epithelial cells and alveolar epithelia (Bao et al., 2020). SARS-CoV-2 infections induced host immune responses and specific anti-viral antibodies would be detected in individuals with exposure. Detection of specific antibodies against SARS-CoV-2 is a useful tool to help find much more asymptomatic infection and past infection, which is effective supplement of nucleic acid testing and a practical way in retrospective survey.

Since it has always been very hard and complicated to determine the origins of novel pathogens (Wang et al., 2021), Chinese scientists have called international cooperation and integrative investigations worldwide to find clues for the origins of SARS-CoV-2 (Wu et al., 2021). Several countries detected samples from sewage (Fongaro et al., 2021), stored in past study (Apolone et al., 2021) or from surveillance for influenza-like illness (Kong et al., 2020), collected before the time when circulation of the virus. In addition, blood donor-based archived specimens are increasingly recognized as great value to retrospectively monitor infectious diseases especially emerging infectious diseases (Busch et al., 2021). In our previous study, we tested 38,144 plasmas collected from blood donors donated during January to April 2020 in Wuhan, Shenzhen, and Shijiazhuang in China and found 395 of 17,794 contained neutralizing antibodies (NAbs) against SARS-CoV-2 in Wuhan. The first seropositive sample was donated on 20 January 2020 (Chang et al., 2021). According to the report of WHO-convened Global Study of Origins of SARS-CoV-2: China part (World Health Organization, 2021), the international joint team recommends a serosurvey for SARS-CoV-2 in Wuhan or other locations world-wide using the blood samples from adult blood donors collected at least 3–4 months before the virus circulation. Therefore, to further explore whether there is any evidence on the occurrence of SARS-CoV-2 before December 2019 in China, we here evaluated specific antibodies against SARS-CoV-2 on all available donation samples donated during 1 September to 31 December 2019 in Wuhan Blood Center.

## Results

### Characteristics of enrolled blood donors and blood donations

A total of 76,844 blood donors donated blood in Wuhan between 1 September to 31 December 2019, and we obtained 88,517 blood donation samples from these donors, including 68,456 whole blood donations and 20,061 platelet donations. Of these, 44,667 donation samples were unqualified for further testing. Finally, 32,484 blood donors and their 43,850 blood donation samples were enrolled in the study. The characteristics of total and involved donors are summarized in Table 1 and the two groups showed no significant difference on all the collected characteristics except the sex. The median age of involved blood donors was 20 [interquartile range (IQR), 19–31]. Among all these enrolled donors, 46.9% were female.

**Table 1.** Characteristics of blood donors donated blood during September to December 2019 in Wuhan.

	Total (*n* = 76,844)	Involved blood donors (*n* = 32,484)
No. of donations	88,517	43,850
Whole blood	68,456	23,799
Platelet	20,061	20,051
Sex (%)		
Male	47,621 (62.0)	17,250 (53.1)
Female	29,223 (38.0)	15,234 (46.9)
Age (%)		
Median (IQR, year)	21 (19–29)	20 (19–31)
18–25	53,318 (69.4)	22,557 (69.4)
26–35	12,777 (16.6)	5010 (15.4)
36–45	7,007 (9.1)	3,093 (9.5)
46–55	3,569 (4.6)	1,715 (5.3)
>55	173 (0.2)	109 (0.3)
ABO blood type (%)		
A	24,816 (32.3)	10,481 (32.3)
B	18,692 (24.3)	7,998 (24.6)
O	26,352 (34.3)	10,913 (33.6)
AB	6,984 (9.1)	3,092 (9.5)
Ethnicity (%)		
Han	70,397 (91.6)	29,787 (91.7)
Non-Han	5,449 (7.1)	2,349 (7.2)
Missing data	998 (1.3)	348 (1.1)
Occupation (%)		
Student	44,803 (58.3)	19,540 (60.2)
Freelancer	8,836 (11.5)	3,520 (10.8)
Office worker	6,757 (8.8)	3,009 (9.3)
Worker	3,848 (5.0)	1,427 (4.4)
Business and service personnel	2,589 (3.4)	1,059 (3.3)
Civil worker/teacher/healthcare worker	1,645 (2.1)	693 (2.1)
Farmer	995 (1.3)	312 (1.0)
Military personnel	654 (0.9)	213 (0.7)
Others	5,212 (6.8)	1,986 (6.1)
Missing data	1,505 (2.0)	725 (2.2)
Education level (%)		
Master/doctorate	1,884 (2.5)	769 (2.4)
Bachelor	35,040 (45.6)	15,272 (47.0)
College	21,335 (27.8)	8,963 (27.6)
High school	10,896 (14.2)	4,185 (12.9)
Lower than high school	5,065 (6.6)	2,066 (6.4)
Others	385 (0.5)	208 (0.6)
Missing data	2,239 (2.9)	1,021 (3.1)

**Figure 1.**
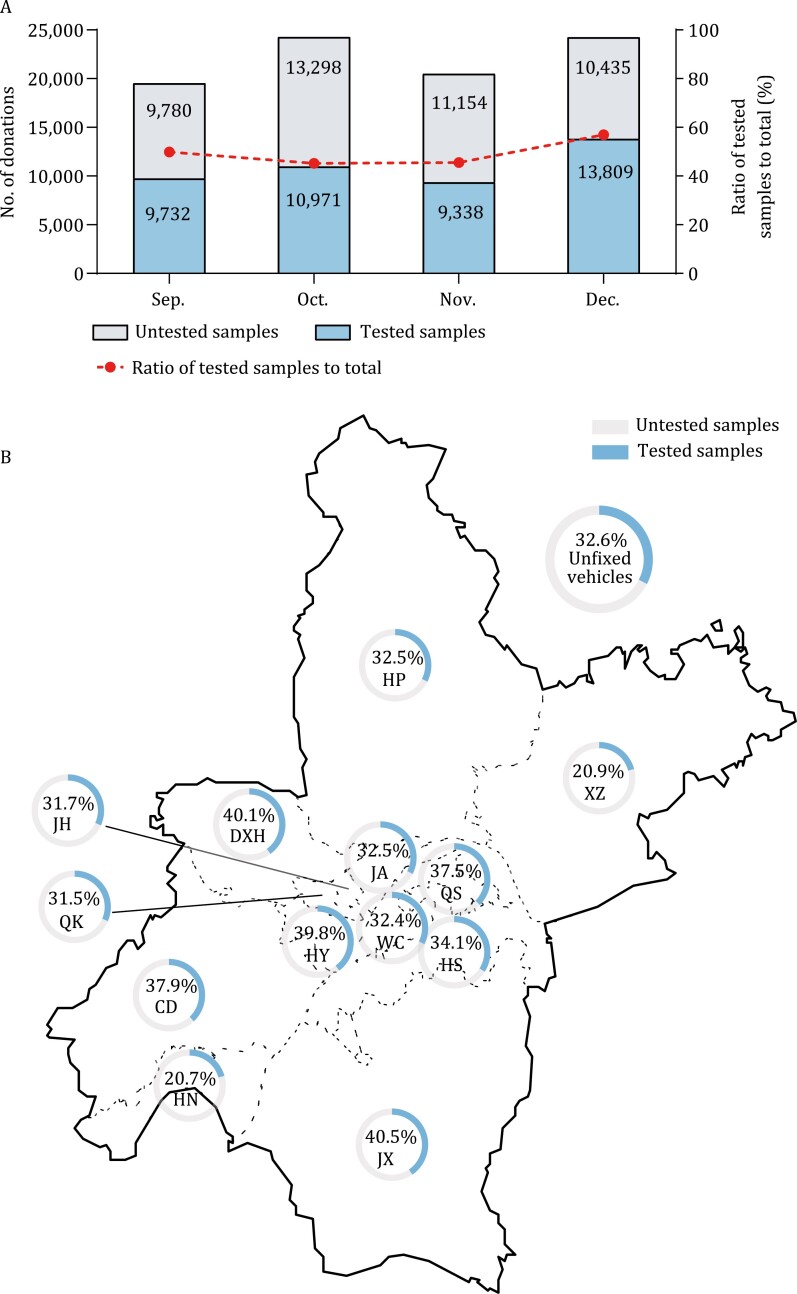
Distribution of donation time and donation sites of involved samples. (A) The number of tested and untested samples were shown in the colored bar. The ratio of tested samples to all donation samples were 49.88% (9732/19 512) in September, 45.21% (10 971/24 269) in October, 45.57% (9338/20 492) in November, and 56.96% (13 809/24 244) in December, respectively. (B) There were over 80 blood collection sites covered all 13 districts in Wuhan. Because apheresis platelets were only collected in two fixed blood-collecting sites, blood donation sites of 23 799 whole blood donors were assessed. The ratio of involved whole blood samples to the total were shown by 14 pie charts, which varied from 20.7% to 40.5%. CD, Caidian Dis.; DXH, Dongxihu Dis.; HN, Hannan Dis.; HP, Huangpi Dis.; HS, Hongshan Dis.; HY, Hanyang Dis.; JA, Jiang’an Dis.; JH, Jianghan Dis.; JX, Jiangxia Dis.; QK, Qiaokou Dis.; QS, Qingshan Dis., WC, Wuchang Dis.; XZ, Xinzhou Dis.

In terms of time distribution, the donation dates of involved blood donations were almost evenly distributed in every month and the ratio of tested donation samples to total samples varied from 45.21% to 56. 96% (Fig. 1A). Besides, we analyzed the distribution of blood donation sites of tested donations. There were more than 80 blood donation sites covering all regions in Wuhan City, including fixed blood-collecting houses, fixed or unfixed blood-collecting vehicles, and sites in colleges and universities. Because apheresis platelets were only collected in two fixed blood-collecting sites, blood donation sites of whole blood donors were assessed. Fig. 1B shows that blood collection sites of 23,799 involved whole blood donations covered all the 13 districts and the proportion of involved samples to total donations were from 20.7% to 40.5%.

### Specific antibodies to SARS-CoV-2 among blood donors

**Figure 2.**
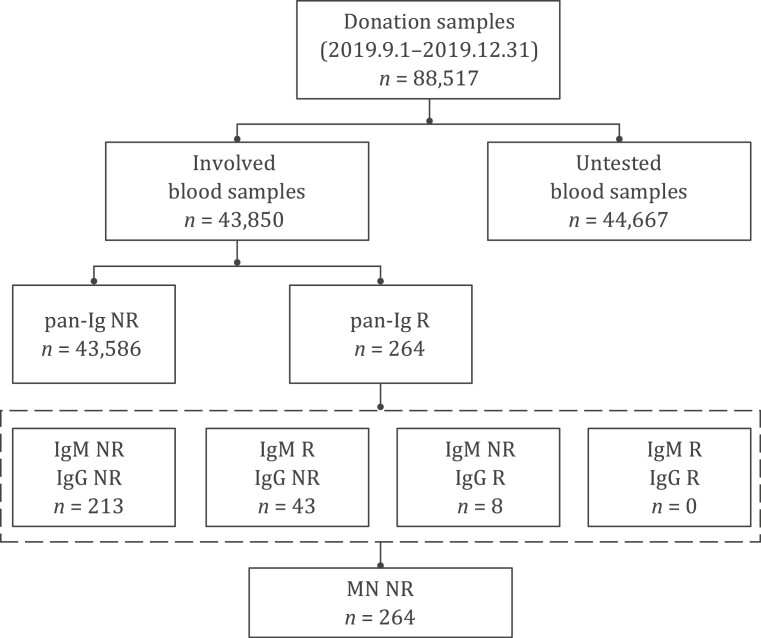
Flow chart of screening and confirmatory procedure and results. A total of 88 517 blood donations were donated between 1 September and 31 December 2019 in Wuhan. Of these, 43 850 achieved samples from 32 484 blood donors were qualified for further pan-Ig testing. 264 pan-Ig reactive samples from 213 blood donors were further tested IgG and IgM antibodies to SARS-CoV-2 and we found 51 reactive (from 38 blood donors): 8 were IgG reactive and 43 were IgM reactive. These 264 samples were finally confirmed negative by microneutralization assay. Neutralizing antibody titers of all samples were <1:8. R, reactive; NR, non-reactive; pan-Ig, pan-immunoglobulins to SARS-CoV-2; IgG, IgG antibody against receptor-binding domain (RBD) of the spike protein of SARS-CoV-2; IgM, IgM antibody against RBD antigen of SARS-CoV-2; MN, microneutralization assay.

After checking the status and volume of achieved samples, 43,850 plasma samples were available to be tested pan-immunoglobulins to SARS-CoV-2 (pan-Ig) in the study. Of these, 264 (264/43,850, 0.602%) donations from 213 (213/32,484, 0.656%) blood donors were pan-Ig reactive. All the reactive samples were further tested for SARS-CoV-2 IgG and IgM, showing that 51 samples from 38 blood donors were IgG or IgM reactive: 8 samples (from 4 donors) were pan-Ig and IgG reactive and 43 (from 34 donors) were pan-Ig and IgM reactive; No samples were both IgG and IgM reactive. Microneutralization assay was performed on all these 264 pan-Ig reactive samples, and all showed negative results (neutralizing antibody titers of all samples were <1:8). The screening and confirmatory procedures and results are shown in Fig. 2.

### Analysis of pan-Ig reactive blood donors

Since no confirmed positive samples were found in microneutralization assay, reactivity of pan-Ig from 264 samples should be false-positive results with the false-positive rate of 0.602% (264/43,850). Therefore, we further analyzed the characteristics of the 213 blood donors and these 264 samples. Above all, we used univariate logistic regression to analyze the difference in the distribution of age, sex, etc. between 213 blood donors with primary screening reactivity of total antibodies to SARS-CoV-2 and all tested blood donors. We found that there was no difference in the distribution of sex, ABO blood type, ethnicity, occupation, and education level (*P* > 0.05). While donors in age group of 46–55 years showed significant difference proportion compared with that in age group of 18–25 (*P* = 0.038 among all groups, *P* = 0.003 between the two groups), suggesting a 2.0-fold [95% confidence interval (95% CI), 1.3–3.2] risk of reactive signal of pan-Ig against SARS-CoV-2.

**Figure 3.**
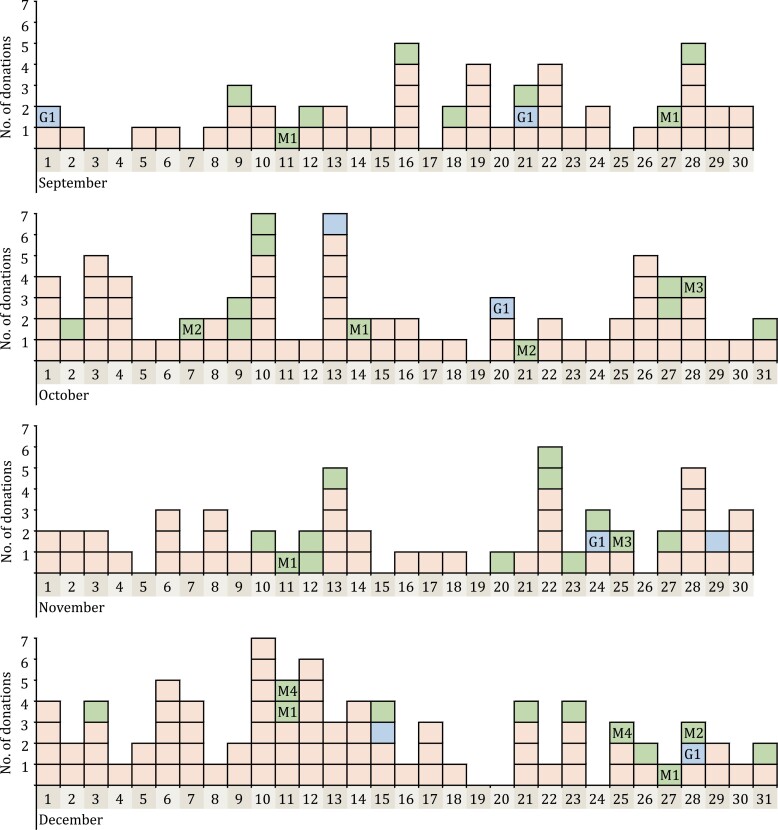
Distribution of donation dates of 264 pan-Ig reactive samples during the four months in 2019. Every colored box showed a donation sample with pan-Ig reactive results. Red box means the sample was only pan-Ig reactive (*n* = 213); Blue box and green box means that not only pan-Ig but also IgG (*n* = 8) or IgM antibodies (*n* = 43) to SARS-CoV-2 was reactive. Blue box with G1 means these five IgG-reactive samples belonged to the same repeat donor named G1. Green box with M1–M4 means the 13 IgM-reactive samples belonged to four repeat donors named M1–M4.

Secondly, the donation time of the 264 pan-Ig reactive samples showed that the false-reactive rate in each month in 2019 was similar, varied from 0.534% to 0.675% (see Table S1). Moreover, donation dates of these samples distributed on nearly every day among the 4 months (Fig. 3). These results both suggested that there was no significant increasing of samples with screening-reactive antibodies to SARS-CoV-2 from September to December 2019. In terms of geographical distribution, donation sites of 161 pan-Ig reactive whole blood donation samples covered 12 of 13 districts in Wuhan City and we did not find a significantly increasing of the reactive rate of pan-Ig in these different regions (see Table S2). Especially, the rate in Jianghan district, where the Huanan Seafood Wholesale Market located, was as low as 0.822% (26/3,164).

Besides, among 264 pan-Ig reactive samples, 77 were from 26 repeat donors, 13 donors of which showed persistently reactive (≥ three times) for pan-Ig, IgG, or IgM antibodies to SARS-CoV-2 during the study (see Table S3). To further explore the true status of these 213 blood donors, we reviewed the SARS-CoV-2 antibodies results of their samples donated from January to June 2020 in our previous study using the same serological screening kits (Chang et al., 2021). Twenty five of 213 blood donors donated blood in Wuhan during the first 6 months in 2020, and samples from 14 donors in 2020 also showed reactive results with similar signal to cutoff ratio (S/CO) values (see Table S3). While samples from other 11 donors were all non-reactive for pan-Ig in 2020. In addition, we did not observe the variations of antibody levels for more than 4-fold. Such findings even more confirmed false-positive results of antibodies to SARS-CoV-2 in serological tests.

## Discussion

Our report is the first large-scale respective study for SARS-CoV-2 specific antibodies among blood donors in Wuhan before 2020. The Coronavirus Disease 2019 (COVID-19) was first identified through a surveillance mechanism for “pneumonia of unknown etiology” in December 2019 in Wuhan, China (Ren et al., 2020; Zhu et al., 2020). Following investigation indicated that the human-to-human transmission has occurred among close contacts since the early December 2019 (Li et al., 2020). However, the exact time of circulation of SARS-CoV-2 in Wuhan is still unknown, which limited our understanding on the origin of the COVID-19 pandemic. In this study, we enrolled 43,850 samples from 32,484 blood donors in Wuhan, donated before or during the initial stage of this outbreak. It clearly showed that no evidence of transmission of COVID-19 before December 2019 in Wuhan, China.

Our previous study also showed that no indication of earlier SARS-CoV-2 circulation and the first confirmed-positive donors donated on 20 January 2020 in Wuhan (Chang et al., 2021). Though 17,794 blood donors in Wuhan were included in that report, only 2,301 donors donated blood in the first 5 weeks in 2020 due to the pandemic, thus the potential limited infected cases or clusters in the initial stage of the outbreak might still be missed (World Health Organization, 2021). Therefore, in current study, we investigated and screened all available stored blood donation samples in Wuhan City through 1 September to 31 December 2019. A total of 43,850 samples, 49.5% (43,850/88,517) of total stored blood donation samples, and 32,484 blood donors, 42.3% (32,484/76,844) of all blood donors who donated during the study period in Wuhan, were tested. The rest 44,667 samples were not accessible due to insufficient volume for automated serological testing, fibrin clot, or severe hemolysis. We analyzed the characteristics between all enrolled donors and total and found nearly no significant difference except the sex (Table 1). In our opinion, due to the higher hematocrit (HCT) of men than women, the volume of plasma from male blood donors may be less than female in the stored pack tube, leading to more male samples with insufficient volume for further testing. These involved samples collected from over 80 donation sites covered all 13 districts of Wuhan City, especially, 3,164 samples were collected from Jianghan district, where the Huanan Seafood Wholesale Market located. In addition, chronologically, 68.5% (30,041/43,850) samples were obtained before December 2019. Considering the representativeness of blood donors and the distribution of donation dates (Fig. 1A) and donation sites between involved samples and the total (Fig. 1B), our study greatly reduced the bias of sampling, achieving a well representation of the true status of seroprevalence of SARS-CoV-2 among blood donors in Wuhan during the study period.

Multiple serological testing and screening approaches were widely used in previous studies, including lateral flow assay (LFA), ELISA, chemiluminescence immunoassay (CLIA), and electrochemiluminescence immunoassay (ECLIA). A serosurvey in Denmark tested IgG and IgM antibodies to SARS-CoV-2 in 20,640 blood donors using LFA method and showed that 412 samples were reactive. After specificity analysis of the method by testing donation samples donated before the occurrence of the virus in Denmark, they found a false-positive rate of 0.46% and adjusted the final seroprevalence results from 2.0% to 1.9% (Erikstrup et al., 2021). Another research among general population in Wuhan enrolled 9,542 individuals from 3,556 families in April 2020 and first screened for pan-Ig with ECLIA kit, then followed by antibody typing testing with in-house ELISAs. 532 (5.6%) were reactive for pan-Ig while only 39.8% contained NAbs confirmed by microneutralization assay as used in this study (He et al., 2021). Our previous study also showed that referring to results of neutralization assays, the false-positive ratio of pan-Ig screening ELISA kits varied from 0.37% to 0.39% (Chang et al., 2021). Therefore, appropriate tests are required to exclude the false-positive reaction derived from immunoassays that may over-estimate the real infection status, particularly for serological studies in a low-prevalence area. In the present study, a combined strategy was employed as before: ELISA kits for specific antibodies screening and microneutralization assay with cultured SARS-CoV-2 used to identify potential positive samples, both methods had been used and proved high sensitivity and specificity in several previous reports (Gudbjartsson et al., 2020; Zhao et al., 2020; Chang et al., 2021; He et al., 2021; Ren et al., 2021).

We here found 264 pan-Ig reactive samples from 213 blood donors without neutralizing antibodies to SARS-CoV-2, with the specificity of 99.40% (43,586/43,850), similar to the reported specificity of Wantai pan-Ig assay (GeurtsvanKessel et al., 2020). These samples were collected and scattered evenly in each month during the study period (Table S1; Fig. 3) and in donation sites from each district in Wuhan City (Table S2). Following analysis on their donation samples from September 2019 to June 2020 showed that samples donated from one repeat donor could achieve persistent reactive results (Table S3). We supposed that some potential nonspecific antibodies or some components from these donors may interfere with the detection of antibodies to SARS-CoV-2. It has been proved that SARS-CoV-2 shared some immunity epitopes with SARS-CoV (Lv et al., 2020; Pinto et al., 2020; Yuan et al., 2020). What’s more, preexisting memory CD4+ T cells, getting from infection by the common human coronaviruses, including HCoV-OC43, HCoV-229E, HCoV-NL63, and HCoV-HKU1, could realize cross-reactive with comparable affinity to SARS-CoV-2 (Mateus et al., 2020; Post et al., 2020). These cross-reactivity usually showed in antibody-binding response, while cross-neutralization activities are rare (Post et al., 2020). Antibodies against Dengue virus also showed cross-reactivity with SARS-CoV-2 (Lustig et al., 2021). Besides, some components in the blood circulation, such as rheumatoid factor, heterophilic antibody, as is known to all, could cause a false-reactive results in antibody detection. The analysis of difference in distribution of characteristics between 213 primary pan-Ig reactive blood donors and all tested donors also supported the suppositions above: since more nonspecific antibodies against other pathogens or components in the circulation may appear in older blood donors than younger donors, thus the reactive proportion of pan-Ig was relatively higher in donors aged from 46 to 55 years old (Table 2).

**Table 2.** Difference in the distribution of characteristics between all tested blood donors and 213 pan-Ig reactive donors.

	pan-Ig reactive donors (prevalence %)	OR (95% CI)^a^	*P* value
Sex (%)			0.210
Male	104 (0.60)	1.0	
Female	109 (0.72)	1.2 (0.9–1.6)	
Age (%)			0.038
Median (IQR, year)	21 (19–32)		
18–25	137 (0.61)	1.0	
26–35	37 (0.74)	1.2 (0.8–1.8)	0.289
36–45	17 (0.55)	0.9 (0.5–1.5)	0.696
46–55	21 (1.22)	2.0 (1.3–3.2)	0.003
>55	1 (0.92)	1.5 (0.2–10.8)	0.687
ABO blood type (%)			0.359
A	69 (0.66)	1.0	
B	46 (0.58)	0.9 (0.6–1.3)	0.477
O	82 (0.75)	1.1 (0.8–1.6)	0.417
AB	16 (0.52)	0.8 (0.5–1.4)	0.384
Ethnicity (%)			0.507
Han	200 (0.67)	1.0	
Non-Han	11 (0.47)	0.7 (0.4–1.3)	0.264
Missing data	2 (0.57)	0.8 (0.2–3.1)	0.720
Occupation (%)			0.170
Student	113 (0.58)	1.0	
Freelancer	26 (0.74)	1.3 (0.8–2.0)	0.259
Office worker	25 (0.83)	1.4 (0.9–2.2)	0.100
Worker	5 (0.35)	0.6 (0.2–1.5)	0.272
Business and service personnel	8 (0.76)	1.3 (0.6–2.7)	0.464
Civil worker/teacher/healthcare worker	5 (0.72)	1.2 (0.5–3.1)	0.627
Farmer	0 (0.00)	0.0 (0.0)	0.994
Military personnel	2 (0.94)	1.6 (0.4–6.6)	0.496
Others	22 (1.11)	1.9 (1.2–3.0)	0.005
Missing data	7 (0.97)	1.7 (0.8–3.6)	0.187
Education level (%)			0.149
Master/doctorate	5 (0.65)	1.0	
Bachelor	83 (0.54)	0.8 (0.3–2.1)	0.696
College	60 (0.67)	1.0 (0.4–2.6)	0.950
High school	34 (0.81)	1.3 (0.5–3.2)	0.641
Lower than high school	19 (0.92)	1.4 (0.5–3.8)	0.488
Others	1 (0.48)	0.7 (0.1–6.4)	0.782
Missing data	11 (1.08)	1.7 (0.6–4.8)	0.347

^a^Odds ratio.

Based on our overall reliable sampling, and screening-confirming testing strategy, we did not identify any blood donors with SARS-CoV-2 NAbs in Wuhan during the study period. Heretofore, using stored blood samples, many countries adopted similar screening-confirming testing strategy. In the United States, a serological survey of 7,389 archived donated blood samples, collected in nine states from 13 December 2019 to 17 January 2020, and identified 106 pan-Ig reactive samples. However, different from our study, they did not verify all these reactive samples, finally 84 of 90 available sera were confirmed to have neutralizing activity (Basavaraju et al., 2021). In Irish blood donors, 8,509 blood samples were tested by three chemiluminescence reagents and then 2.2% were confirmed positive for the presence of SARS-CoV-2 antibodies by the same pan-Ig assays in the present study (Butler et al., 2022). In Italy, researchers found SARS-CoV-2 RBD-binding antibodies in 111 of 959 (11.6%) individuals, and six were finally confirmed by a qualitative microneutralization assay, starting from October 2019 (Apolone et al., 2021).

Not only for respective studies on SARS-CoV-2, blood donor-based archived samples were also usually used to study the origin of other emerging infectious diseases and monitor the incidence among local population, and Zika virus is a good example. Zika virus was identified in the year of 1947 in the forest of Uganda (Dick et al., 1952) and there were only some small-scale outbreaks in Africa and Asia before 2015. While it caused a major outbreak since May 2015 in Brazil, then fast spread over all America. However, a study showed that blood donor samples collected in March 2015 from the state of São Paulo, the southeast region of Brazil, was positive for the nucleic acid of the virus (Slavov et al., 2020). Similarly, in Colombia, the first registered confirmed Zika virus infected case was reported in October 2015. Samples from local blood banks before the date were tested to estimate the presence of the virus in Colombia. Researchers found samples from June to July 2015 contained antibodies against Zika virus and the seroprevalence grown over time (Bayona-Pacheco et al., 2019), which suggesting that the occurrence of the virus were much earlier than reported.

However, compared with general population, it should be noted that there are differences in the distribution of demographic characteristics of blood donors. For example, only healthy people aged 18–55 years could donate blood in China and the condition can be relaxed to 60 years old for repeat donors. Besides, since there are many colleges and universities in Wuhan City, students are main group of blood donors, thus in the study the median age of involved and total donors was only 20 and 21 years old, respectively. In addition, “healthy donor effect” should be considered, as people with mild illness or discomfort are not included (Atsma et al., 2011). Besides, due to 14% preservative fluid in the pack tubing plasma, we set the cutoff ratio (S/CO) of serological tests to 0.8 in the study, which may increase the sensitivity of screening test but bring more false reactivities.

In summary, the respective serosurvey of SARS-CoV-2 enrolled 32,484 blood donors and tested 43,850 samples collected from 1 September to 31 December 2019 and showed no specific SARS-CoV-2 antibodies among blood donors in Wuhan, China, indicating that no evidence of transmission of COVID-19 before December 2019 in Wuhan, China.

## Materials and methods

### Study design and participants

Blood donation samples donated from 1 September to 31 December 2019 in Wuhan Blood Center were all enrolled in the study. Anonymous personal demographic information from blood donors, including gender, age, ethnicity, occupation, and educational level, and blood type were collected. All the long-striped archived blood specimens, which connected with the blood bag during blood collection, had been stored under −20°C at least 2 years after the blood products were used, which aimed to deal with possible legal disputes caused by blood transfusion according to relevant Chinese regulations. These archived plasma samples were centrifuged and transported to clean tubes, and samples with volume of <300 μL (considering the minimum volume for antibody testing and the loss of transfer process), fibrin clot, severe hemolysis were unincluded for further testing.

### Serological tests

All the available and qualified plasma samples were screened for pan-immunoglobulins to SARS-CoV-2 (pan-Ig), and reactive samples were further tested for SARS-CoV-2 specific IgG and IgM antibodies in twice (Wantai, Beijing, China). All the serological screening tests were enzyme-linked immunosorbent assay (ELISA) method as previously reported (Chang et al., 2021). In brief, pan-Ig detection was based on a double antigens sandwich immunoassay using recombinant antigens contained the receptor-binding domain (RBD) of the spike protein of SARS-CoV-2. IgG antibody was tested using an indirect ELISA method with recombinant RBD antigen, and IgM antibody was tested by μ-chain capture method with recombinant RBD antigen. Since there was approximately 14% preservative fluid in the pack tubing plasma, the cutoff ratio (S/CO) of serological tests was set as 0.8. A study based-on cross-assay comparisons in parallel demonstrated that the total antibodies ELISA outperformed all other assays that only detected single antibody isotype (GeurtsvanKessel et al., 2020). In addition, the reported sensitivity of Wantai pan-Ig ELISA assay was 100% since 2 weeks after onset (Lou et al., 2020; Zhao et al., 2020), and the specificity was ranging from 99.3% to 100% (GeurtsvanKessel et al., 2020; Lassaunière et al., 2020).

### Microneutralization assay

Samples with reactive pan-Ig antibodies results were further verified by analysis of neutralizing antibodies to SARS-CoV-2, which were assessed at Institute of Pathogen Biology, Chinese Academy of Medical Sciences and Peking Union Medical College, Beijing, China by using microneutralization assay as previously reported (He et al., 2021; Ren et al., 2021). Serially 2-fold diluted plasma (from 1:4 to 1:128) were mixed with equal volumes of SARS-CoV-2 (IPBCAMS-WH-01/2019, EPI_ISL_402123) with 100 50% tissue culture infective doses (TCID50) and preincubated at 37°C for 1 h. The virus/plasma mixture were incubated with Vero cells (ATCC CCL-81) in 96-well plates (Corning, NY, USA) at 37°C for 1 h, then replaced with fresh culture medium. The cytopathic effect was observed 5 days after incubation. Four duplicate wells were used for each plasma dilution. The neutralizing effects were determined by using Reed-Muench method (REED et al., 1938). Viral back-titration was done, and plasma samples known to be positive and negative for neutralizing antibodies were used as positive control and negative control in each test. The cutoff for a positive neutralizing antibody titer was 1:8.

### Statistical analysis

The difference of demographic characteristic between all tested blood donors and 213 blood donors with reactive pan-Ig against SARS-CoV-2 were estimated by univariate logistic regression. A *P* value less than 0.05 was considered statistically significant. All the data were collected via Microsoft Excel 365 (Microsoft Corporation by Impressa Systems, Santa Rosa, CA) and the statistical analyses above were realized by SPSS v21.0 (IBM SPSS, Chicago, IL).

## Supplementary Material

pwac013_suppl_Supplementary_Material

## Data Availability

All data generated or analyzed during this study are included in this published article (and its supplementary material file).
