# Supplementary material for: Serosurvey for SARS-CoV-2 among blood donors in Wuhan, China from September to December 2019
Source: Protein Cell. 2022 May 24;14(1):28–36. doi: 10.1093/procel/pwac013 (PMC9871965; doi:10.1093/procel/pwac013)
Supplement: pwac013_suppl_Supplementary_Material [file pwac013_suppl_supplementary_material.docx]

**Supplementary Material for**

**Serosurvey for SARS-CoV-2 among blood donors in Wuhan, China from September to December 2019**

Le Chang^1,2†^, Lei Zhao^3,4†^, Yan Xiao^5,6^, Tingting Xu^3,4^, Lan Chen^5,6^, Yan Cai^7^, Xiaojing Dong^5,6^, Conghui Wang^5,6^, Xia Xiao^5,6^, Lili Ren^5,6 *^, Lunan Wang^1,2,8 *^

1. National Center for Clinical Laboratories, Beijing Hospital, National Center of Gerontology; Institute of Geriatric Medicine, Chinese Academy of Medical Sciences, Beijing, P.R. China

2. Beijing Engineering Research Center of Laboratory Medicine, Beijing Hospital, Beijing, P.R. China

3. Department of Laboratory, Wuhan Blood Center, Wuhan, P.R. China

4. Confirmation Laboratory for Transfusion Transmitted Disease, Institute of Blood Transfusion of Hubei Province, Wuhan, P.R. China

5. National Health Commission of the People’s Republic of China Key Laboratory of Systems Biology of Pathogens, Institute of Pathogen Biology, Chinese Academy of Medical Sciences and Peking Union Medical College, Beijing, P.R. China.

6. Key Laboratory of Respiratory Disease Pathogenomics, Chinese Academy of Medical Sciences and Peking Union Medical College, Beijing, P.R. China.

7. Department of Quality Control, Wuhan Blood Center, Wuhan, P.R. China

8. Graduate School, Peking Union Medical College, Chinese Academy of Medical Sciences, Beijing, P.R. China

† These authors contributed equally to this article.

*Corresponding author. Email: renliliipb@163.com (L.L.R.); lunan99@163.com (L.N.W.)

Table S1. False positive rates of pan-Ig to SARS-CoV-2 from September to December 2019

| Mon | No. of involved donation samples | No. of pan-Ig reactive samples | false positive rate of pan-Ig (%) | No. of IgG or IgM reactive samples | false positive rate of IgG or IgM (%) |
| --- | --- | --- | --- | --- | --- |
| Sep. | 9,732 | 52 | 0.534 | 10 | 0.103 |
| Oct. | 10,971 | 74 | 0.675 | 14 | 0.128 |
| Nov. | 9,338 | 56 | 0.600 | 14 | 0.150 |
| Dec. | 13,809 | 82 | 0.594 | 13 | 0.094 |
| Total | 43,850 | 264 | 0.602 | 51 | 0.116 |

Table S2. False positive rates of 161 pan-Ig reactive whole blood donation samples in 13 districts in Wuhan City

|  | Districts in Wuhan | No. of involved donation samples | No. of pan-Ig reactive samples | false positive rate of pan-Ig (%) |
| --- | --- | --- | --- | --- |
| urban  area | Hongshan Dis. | 7,964 | 47 | 0.590 |
|  | Wuchang Dis. | 3,933 | 29 | 0.737 |
|  | Jianghan Dis. | 3,164 | 26 | 0.822 |
|  | Hanyang Dis. | 1,185 | 6 | 0.506 |
|  | Qiaokou Dis. | 490 | 1 | 0.204 |
|  | Jiangan Dis. | 150 | 1 | 0.667 |
|  | Qingshan Dis. | 99 | 1 | 1.010 |
| suburb area | Jiangxia Dis. | 4,201 | 25 | 0.595 |
|  | Dongxihu Dis. | 1,098 | 8 | 0.729 |
|  | Caidian Dis. | 467 | 7 | 1.499 |
|  | Huangpi Dis. | 150 | 1 | 0.667 |
|  | Hannan Dis. | 37 | 1 | 2.703 |
|  | Xinzhou Dis. | 31 | 0 | 0.000 |
|  | Unfixed blood-collecting vehicle | 830 | 8 | 0.964 |
| Total number of whole blood donations | | 23,799 | 161 | 0.676 |

Table S3. Serological results of repeat donors donated from September 2019 to June 2020

| No. of donor | donation date | pan-Ig (S/CO) | IgG (S/CO) | IgM (S/CO) |
| --- | --- | --- | --- | --- |
| 0027 | 2019-9-11 | 2.005 | NR | 2.62 |
|  | 2019-9-27 | 2.147 | NR | 2.87 |
|  | 2019-10-14 | 1.737 | NR | 2.3 |
|  | 2019-11-11 | 3.274 | NR | 2.36 |
|  | 2019-12-11 | 4.184 | NR | 3.89 |
|  | 2019-12-27 | 2.347 | NR | 2.92 |
| 6310 | 2019-10-15 | 1.126 | NR | NR |
|  | 2019-12-6 | 1.053 | NR | NR |
| 061X | 2019-9-13 | 3 | NR | NR |
|  | 2019-10-3 | 4.663 | NR | NR |
|  | 2019-11-2 | 2.29 | NR | NR |
| 0011 | 2019-9-8 | 1.911 | NR | NR |
|  | 2019-9-28 | 1.168 | NR | NR |
|  | 2019-10-13 | 1.232 | NR | NR |
|  | 2019-10-27 | N/A |  |  |
|  | 2019-11-16 | 0.984 | NR | NR |
|  | 2019-12-3 | 0.879 | NR | NR |
|  | 2019-12-17 | N/A |  |  |
| 0015 | 2019-12-15 | 0.937 | 1.04 | NR |
|  | 2019-12-29 | NR |  |  |
| 0093 | 2019-10-20 | 0.821 | NR | NR |
|  | 2019-12-22 | NR |  |  |
| 1712 | 2019-9-2 | 0.874 | NR | NR |
|  | 2019-10-17 | NR |  |  |
| 1016 | 2019-9-13 | NR |  |  |
|  | 2019-11-12 | 2.411 | NR | 1.21 |
| 5673 | 2019-9-8 | NR |  |  |
|  | 2019-9-28 | NR |  |  |
|  | 2019-10-12 | 0.816 | NR | NR |
|  | 2019-10-26 | 1.479 | NR | NR |
|  | 2019-11-9 | NR |  |  |
|  | 2019-12-1 | 1.142 | NR | NR |
|  | 2019-12-30 | NR |  |  |
| 0038 | 2019-9-16 | 2.221 | NR | NR |
|  | 2019-10-10 | 1.99 | NR | NR |
|  | 2019-10-26 | 2.547 | NR | NR |
|  | 2019-11-27 | 2.484 | NR | NR |
|  | 2019-12-11 | 1.89 | NR | NR |
| 4267 | 2019-10-28 | 1.416 | NR | 1.58 |
|  | 2019-11-25 | 2.105 | NR | 1.14 |
| 0418 | 2019-9-1 | 8.953 | 16.19 | NR |
|  | 2019-9-21 | 7.147 | 16.33 | NR |
|  | 2019-10-20 | 6.153 | 16.38 | NR |
|  | 2019-11-24 | 8.016 | 15.33 | NR |
|  | 2019-12-28 | 5.8 | 16.06 | NR |
| 0347 | 2019-10-20 | 1.6 | NR | NR |
|  | 2019-12-14 | 1.147 | NR | NR |
| 5673 | 2019-10-12 | 0.816 | NR | NR |
|  | 2019-10-26 | 1.479 | NR | NR |
|  | 2019-12-1 | 1.142 | NR | NR |
| 5899 | 2019-11-1 | 4.958 | NR | NR |
|  | 2019-11-22 | 4.284 | NR | NR |
| 0069 | 2019-10-10 | 9.39 | NR | NR |
|  | 2019-11-4 | 8.105 | NR | NR |
| 9568 | 2019-9-16 | 1.305 | NR | NR |
|  | 2019-10-28 | 1.221 | NR | NR |
|  | 2019-11-25 | 1.358 | NR | NR |
|  | 2019-12-26 | 1.332 | NR | NR |
|  | 2020-5-2 | 1.232 | NR | NR |
|  | 2020-5-27 | 1.679 | NR | NR |
| 3924 | 2019-9-4 | NR |  |  |
|  | 2019-9-18 | NR |  |  |
|  | 2019-10-20 | NR |  |  |
|  | 2019-11-29 | 2.737 | NR | NR |
|  | 2019-12-14 | 1.3 | NR | NR |
|  | 2020-1-19 | NR |  |  |
|  | 2020-5-9 | NR |  |  |
|  | 2020-5-30 | NR |  |  |
| 1015 | 2019-12-31 | 1.195 | NR | 2.69 |
|  | 2020-5-8 | 1.037 | NR | 1.90 |
| 0817 | 2019-11-28 | 2.716 | NR | NR |
|  | 2020-1-14 | 1.184 | NR | NR |
|  | 2020-4-14 | 1.279 | NR | NR |
| 4033 | 2019-10-10 | NR |  |  |
|  | 2019-12-2 | 0.847 | NR | NR |
|  | 2020-5-12 | NR |  |  |
| 5570 | 2019-9-14 | 2.105 | NR | NR |
|  | 2019-12-21 | 2.426 | NR | NR |
|  | 2020-2-6 | NR |  |  |
|  | 2020-2-20 | NR |  |  |
|  | 2020-3-11 | NR |  |  |
|  | 2020-4-6 | NR |  |  |
|  | 2020-5-10 | NR |  |  |
| 4126 | 2019-12-1 | 2.584 | NR | NR |
|  | 2020-4-3 | NR |  |  |
| 5430 | 2019-9-16 | 7.647 | NR | 2.43 |
|  | 2020-4-7 | 3.879 | NR | 1.41 |
| 5633 | 2019-9-30 | 0.921 | NR | NR |
|  | 2019-10-16 | 0.905 | NR | NR |
|  | 2019-11-5 | NR |  |  |
|  | 2019-12-9 | 0.868 | NR | NR |
|  | 2020-5-7 | NR |  |  |
|  | 2020-5-23 | NR |  |  |
| 6312 | 2019-9-22 | 10.126 | NR | NR |
|  | 2019-11-17 | 5.542 | NR | NR |
|  | 2020-4-19 | 5.716 | NR | NR |
| 1552 | 2019-10-10 | 3.737 | NR | NR |
|  | 2020-4-19 | 1.095 | NR | NR |
| 1023 | 2019-10-5 | NR |  |  |
|  | 2019-10-19 | NR |  |  |
|  | 2019-12-21 | 1.047 | NR | NR |
|  | 2020-2-26 | 1.179 | NR | NR |
|  | 2020-3-27 | NR |  |  |
|  | 2020-5-5 | NR |  |  |
|  | 2020-5-30 | NR |  |  |
| 1617 | 2019-9-19 | 1.842 | NR | NR |
|  | 2019-10-30 | 1.2 | NR | NR |
|  | 2019-11-15 | NR |  |  |
|  | 2019-12-6 | 1.058 | NR | NR |
|  | 2019-12-22 | 0.847 | NR | NR |
|  | 2020-5-24 | NR |  |  |
| 1627 | 2019-10-22 | 0.926 | NR | NR |
|  | 2019-11-14 | NR |  |  |
|  | 2019-12-26 | NR |  |  |
|  | 2020-2-2 | NR |  |  |
|  | 2020-4-9 | NR |  |  |
|  | 2020-5-27 | NR |  |  |
| 6311 | 2019-9-11 | NR |  |  |
|  | 2019-10-30 | NR |  |  |
|  | 2019-11-25 | NR |  |  |
|  | 2019-12-11 | 0.947 | NR | 30.07 |
|  | 2019-12-25 | 0.868 | NR | 24.50 |
|  | 2020-5-22 | NR |  |  |
|  | 2020-6-5 | NR |  |  |
| 3816 | 2019-9-15 | 0.921 | NR | NR |
|  | 2019-10-1 | 1.195 | NR | NR |
|  | 2019-11-9 | NR |  |  |
|  | 2019-12-15 | 1.032 | NR | NR |
|  | 2020-5-4 | 0.995 | NR | NR |
|  | 2020-6-1 | NR |  |  |
| 0416 | 2019-10-1 | 1.553 | NR | NR |
|  | 2019-10-23 | 0.895 | NR | NR |
|  | 2020-1-16 | 1.105 | NR | NR |
|  | 2020-6-4 | NR |  |  |
| 7198 | 2019-10-4 | 4.368 | NR | NR |
|  | 2020-1-20 | 1.626 | NR | NR |
|  | 2020-5-12 | 1.416 | NR | NR |
| 8223 | 2019-9-9 | 2.184 | NR | NR |
|  | 2019-10-8 | 4.421 | NR | NR |
|  | 2020-3-13 | 1.321 | NR | NR |
| 5973 | 2019-9-16 | 4.468 | NR | NR |
|  | 2019-12-1 | 5.605 | NR | NR |
|  | 2020-4-4 | NR |  |  |
| 8557 | 2019-11-3 | 0.995 | NR | NR |
|  | 2019-12-7 | 1.537 | NR | NR |
|  | 2019-12-28 | 1.495 | NR | NR |
|  | 2020-1-20 | 1.926 | NR | NR |
|  | 2020-2-16 | 1.763 | NR | NR |
|  | 2020-3-2 | NR |  |  |
|  | 2020-3-25 | NR |  |  |
|  | 2020-4-11 | 1.432 | NR | NR |
|  | 2020-5-1 | 1.584 | NR | NR |
| 2573 | 2019-10-7 | 2.258 | NR | 4.10 |
|  | 2019-10-21 | 2.811 | NR | 3.65 |
|  | 2019-12-28 | 1.895 | NR | 4.75 |
|  | 2020-1-22 | NR |  |  |
|  | 2020-4-15 | 0.832 | NR | 0.914 |
|  | 2020-5-30 | NR |  |  |
| 0856 | 2019-9-8 | NR |  |  |
|  | 2019-10-13 | 0.932 | NR | NR |
|  | 2019-12-8 | NR |  |  |
|  | 2019-12-29 | NR |  |  |
|  | 2020-4-12 | NR |  |  |
|  | 2020-5-5 | NR |  |  |
|  | 2020-5-31 | 0.847 | NR | NR |
| 001X | 2019-10-2 | 0.9 | NR | NR |
|  | 2019-10-18 | NR |  |  |
|  | 2019-11-24 | 0.868 | NR | NR |
|  | 2019-12-10 | 1.063 | NR | NR |
|  | 2019-12-29 | 0.94 | NR | NR |
|  | 2020-4-5 | NR |  |  |
|  | 2020-5-13 | NR |  |  |
|  | 2020-5-29 | NR |  |  |
| 242X | 2019-9-8 | NR |  |  |
|  | 2019-9-23 | NR |  |  |
|  | 2019-10-7 | NR |  |  |
|  | 2019-10-21 | NR |  |  |
|  | 2019-11-4 | NR |  |  |
|  | 2019-11-18 | NR |  |  |
|  | 2019-12-9 | 0.826 | NR | NR |
|  | 2019-12-23 | 0.958 | NR | NR |
|  | 2020-1-22 | NR |  |  |
|  | 2020-2-11 | NR |  |  |
|  | 2020-2-25 | NR |  |  |
|  | 2020-3-18 | NR |  |  |
|  | 2020-4-1 | NR |  |  |
|  | 2020-5-7 | NR |  |  |
| NR, S/CO<0.8 |  |  |  |  |
| N/A, unavailable |  |  |  |  |
